# Adverse childhood experiences and resting state functional connectivity of the triple brain network: a meta-analysis

**DOI:** 10.1007/s00406-026-02204-2

**Published:** 2026-02-12

**Authors:** Giulia Maria Giordano, Giulia Cattarinussi, Andrew Lawrence, Nare Amasi-Hartoonian, Svenja Kretzer, Xuemei Ma, Rebecca Pollard, Corentin Vallée, Armida Mucci, Silvana Galderisi, Paola Dazzan

**Affiliations:** 1 Department of Mental and Physical Health and Preventive Medicine, School of Medicine, University of Campania Luigi Vanvitelli, Largo Madonna delle Grazie 1, 80135 Naples, Italy; 2 Department of Psychological Medicine, Institute of Psychiatry, Psychology and Neuroscience, King’s College London, 16 De Crespigny Park, London, SE5 8AF UK; 3 NIHR Maudsley Biomedical Research Centre at South London and Maudsley NHS Foundation Trust, London, UK; 4 Singapore Institute for Clinical Sciences, Agency for Science, Technology & Research (A * STAR) Singapore, Singapore, Republic of Singapore

**Keywords:** Adverse childhood experiences, ACEs, Resting-state functional connectivity, rs-fMRI, Meta-analysis

## Abstract

Adverse childhood experiences (ACEs), which include potentially traumatic events occurring during childhood, have been found to be associated with alterations in resting-state functional connectivity (rs-FC) within large brain networks, such as the Salience (SN), Default Mode (DMN), and Central Executive (CEN) Networks. While alterations in these networks may increase vulnerability to mental health disorders, existing data are inconsistent as to the neural pathways affected and the type of connectivity pattern associated with adversity (hyper- or hypoconnectivity). These inconsistencies could be due to methodological differences across studies, to the selection of different target regions of interest (ROIs), or to the use of ROI-to-ROI approaches that could overlook broader effects at the whole-brain level. Here, we conducted a meta-analysis of rs-FC seed-to-whole brain studies using the Seed-based d Mapping software to investigate patterns of rs-FC aberrations specifically associated with ACEs. Out of 12 identified studies, eight were included in the meta-analysis, comprising 289 individuals positive for ACEs and 301 controls. The analysis showed that individuals positive for ACEs, compared to controls, present a pattern of hypoconnectivity centred within the SN, between SN and DMN integrative hubs, and between DMN and CEN. Such disruptions, potentially resulting from prolonged or excessive activity of the hypothalamus-pituitary-adrenal axis, might affect emotion regulation, fear conditioning, memory and affective learning processing, goal-directed behavior, and cognitive control, and increase vulnerability to mental health disorders. Future research should focus on more homogeneous populations and larger samples, to truly advance our understanding of the biological mechanisms underlying ACEs-related brain connectivity disruptions.

**Supplementary Information:**

The online version contains supplementary material available at 10.1007/s00406-026-02204-2.

## Introduction

Adverse childhood experiences (ACEs) are potentially traumatic events that occur during childhood. They include adverse experiences, such as familial separation, serious accidents or injuries, extreme poverty, family stress and maltreatment (including emotional and physical neglect; emotional, physical, and sexual abuse) [1–3]. It is estimated that every year, millions of children suffer from various forms of ACEs, with the global prevalence of maltreatment ranging between 13% and 36% [4, 5].

Converging evidence suggests that ACEs can have a significant impact on personal development, functioning and attachment styles [6]. ACEs increase vulnerability to maladaptive developmental outcomes, including socio-affective functioning, emotion regulation, reward/error processing, and well-being [7, 8]. As such, they are conceptualised as transdiagnostic risk factors for the onset of multiple mental health conditions, including depression, anxiety, post-traumatic stress disorder, substance use disorders, suicidal behavior, and psychosis [7, 9–11].

From a pathophysiological perspective, repeated exposure to ACEs is associated with prolonged or excessive activity of the hypothalamus-pituitary-adrenal axis, the main stress response system [12–14]. This prolonged activation may lead to changes in brain structure and function, particularly in regions with a dense expression of glucocorticoid receptors, such as the medial prefrontal cortex (mPFC), anterior cingulate cortex (ACC), hippocampus, amygdala, and insula [15–17]. Interestingly, these brain regions are also involved in emotion processing, inhibitory control, memory and reward processing, functions which have been reported as impaired in individuals with a history of ACEs [8].

Resting-state functional MRI (rs-fMRI) can help elucidate the patterns of neural reorganization in brain areas that may be specifically associated with exposure to ACEs. This approach estimates functional connectivity (rs-FC), which is the similarity between neuronal activation patterns of brain regions while the participant is awake and does not perform any task [18, 19]. As brain regions can be structurally remote yet functionally highly connected, rs-FC can provide valuable information on brain function, and particularly on the organization of the brain into those functional networks associated with cognition, emotion regulation and behavior [20].

Among the functional networks that have been associated with ACEs, the Salience Network (SN), the Default Mode Network (DMN) and the Central Executive Network (CEN) are of particular interest [21–26]. The SN is mainly centred in the insula and in the dorsal-anterior cingulate cortex (dACC) and, through the integration of sensory, emotional, and cognitive information, contributes to complex brain functions, including communication, social behavior and self-awareness [27]. The DMN, centred in the mPFC and the posterior cingulate cortex (PCC), is mainly involved in the retrieval and manipulation of episodic memories, self-directed thought or introspection and conceiving others’ perspectives [28]. Finally, the CEN, located mainly in the dorso-lateral prefrontal cortex (DLPFC) and posterior parietal cortex, is involved in high-level cognitive functions, such as planning, decision making and the control of attention and working memory [20].

Together, these three functional networks constitute the so-called “triple network” [20], a dysfunction of which has been implicated in the pathophysiology of mental health disorders, such as depression, anxiety and psychosis [20, 29, 30]. Relevant to the experience of ACEs, these disorders are known to also involve aberrant stress system reactivity, altered emotion regulation and self-processing, and are associated with a higher prevalence of ACEs [20, 29, 30]. Investigating the integrity of rs-FC in individuals exposed to ACEs can therefore provide crucial information on the pathways through which ACEs may heighten the risk of maladaptive outcomes, particularly stress-related psychopathology.

To date, however, findings on which neural pathways are affected and what pattern of connectivity alteration (i.e. whether it is hyper-, hypo-connectivity, or more complex patterns) is related to ACEs remain inconsistent. For example, studies have reported both increased and decreased connectivity of circuits involving key regions such as the amygdala, insula, ACC and hippocampus [24, 31–36]. This heterogeneity might be related to differences in sample characteristics, developmental stages, psychiatric comorbidities, methodological approaches, as well as the selection of different target regions of interest (ROIs), or the use of an ROI-to-ROI approach that could lead to overlooking broader effects at the whole-brain level.

However, a growing body of evidence, including some included in this meta-analysis [17, 33, 37–40], converge in reporting reduced connectivity within and between large-scale networks, particularly the default mode network (DMN) and salience network (SN), as well as in their interactions with other large-scale networks. These alterations have been interpreted as reflecting long-lasting disruptions in the integration of cognitive, affective, and self-referential processes following ACEs.

Furthermore, recent attempts to summarise the evidence in this area include two systematic reviews [29, 41], which investigated only specific adversities, and a meta-analysis of amygdala connectivity [42]. In particular, the systematic review by Gerin and colleagues [29] focused on childhood maltreatment and rs-FC, but it did not include other types of ACEs and it did not conduct a quantitative synthesis. The systematic review by McLaughlin et al. [41] provided a broader overview of how threat and deprivation shape neural development, but it did not specifically address functional connectivity or perform a meta-analysis.Finally, the only available meta-analysis to date has focused exclusively on the amygdala as ROI, examining its functional dysconnectivity during task and resting-state conditions in relation to ACEs, without exploring connectivity across multiple large-scale networks [42].

In contrast, here we conducted the first meta-analysis of studies evaluating any type of ACEs in relation to rs-FC across multiple brain regions rather than a single region of interest. Conducting a seed-to-whole brain analyses can provide a more comprehensive understanding of the neural connectivity patterns associated with ACEs than seed-to-ROI framework. We specifically analyzed reports that investigated seed-to-whole brain FC in relation to adverse childhood experiences, focusing on seeds which could be classified into one of the three “triple-network” networks (SN, DMN, or CEN). Although individual studies report mixed directions of effect, the recurrent finding of hypoconnectivity across independent samples [17, 33, 37–40], as outlined above, suggests a potential overarching trend. Therefore, based on previous empirical evidence [17, 33, 37–40], we assumed that the exposure to ACEs would be more consistently associated with hypoconnectivity than hyperconnectivity across large-scale resting-state networks. Importantly, the present meta-analysis was designed to quantitatively evaluate whether such a trend emerges despite the substantial heterogeneity characterizing the existing literature.

.

## Methods

### Selection criteria and search strategy

We registered this meta-analysis with PROSPERO (ID: CRD42024502099). Following the Preferred Reporting Items for Systematic Reviews and Meta-Analyses (PRISMA) guideline [43], three electronic databases (PubMed, Scopus and PsycINFO) were autonomously searched by two researchers (GMG and GC) from inception to 14.10.2025. The search terms used were: (Child OR Childhood OR Children OR “School Age” OR Infant OR Infancy OR Youth OR Adolescent OR Adolescence OR Preschool) AND (adversities OR “Adverse experience” OR ACES OR “Early-life stress” OR ELS OR maltreatment OR abuse OR “early stress” OR “early-life trauma” OR Neglect OR deprivation) AND (fMRI OR “functional MRI”) AND (“resting-state functional connectivity” OR “resting-state” OR “intrinsic connectivity”). In addition, the reference lists of the manuscripts identified, and relevant reviews and meta-analyses, were manually examined to identify additional study.

### Eligibility criteria

The inclusion criteria adhered to the PICO acronym as detailed below:

*Participants*: individuals previously exposed to ACEs and individuals not exposed to ACEs; *Intervention*: not applicable (NA); *Comparison*: participants who had been exposed to ACEs vs. participants who had not been exposed to ACEs; *Outcomes*: rs-FC alterations of either of the three networks (SN, DMN, and CEN) in participants previously exposed to ACEs.

Articles were included if they met the following criteria: published in English in a peer-review journal; full text available; included participants previously exposed to ACEs evaluated with standardized assessment tools and a control group; included more than 10 participants per group; employed seed-to-whole brain rs-FC Magnetic Resonance Imaging (MRI) methods; seeds were within SN, DMN or CEN boundaries; reported Talairach (TAL) or Montreal Neurological Institute (MNI) peak effect coordinates and corresponding *t*, *z*, or *p* values. Articles were excluded if they did not meet the above-mentioned criteria or if peak coordinates and/or *t*, *z*, or *p* values were not available even after contacting the Authors.

### Data extraction and coding

Publications identified through the initial search were imported into Endnote, where they were checked for duplication. Thereafter, two authors (GMG and GC) independently screened the titles and abstracts of each publication. Following screening, the same authors independently extracted study data from the full text. Data extracted are presented in Tables 1 and 2 and Supplementary Materials, and include: sample size, percentage of male and females, mean age, assessment instruments used for the evaluation of ACEs exposure, psychiatric comorbidity, medications, instructions given to participants (eyes open, closed or fixated), magnetic field intensity, seed, functional connectivity model and peak coordinates of each significant between-group effect for each included seed. Where only *z* or *p* values for the effect peaks were available, these were converted into *t* values.

**Table 1:** Demographic and clinical characteristics of the 8 studies including individuals from the general population or individuals recruited from social care

Participants positive for ACEs (*N* = 289)							Participants negative for ACEs (*N* = 301)					
Study	N	Mean age (SD)	Female (%)	Adversities tools and maltreatment types	Psychiatric Comorbidities	Psychiatric Medications	N	Mean age (SD)	Female (%)	Psychiatric Comorbidities	Psychiatric Medications	
Van der Weff et al., 2013 [49]	44	39 (10.30)	50	*NEMESIS trauma interview.* *Emotional neglect*: 97.7%; *Emotional abuse*: 29.5%	MDD 30%; ANX 20%; MDD and ANX 31%	NR	44	37.6 (9.7)	54.5	MDD 43%; ANX 16%; MDD and ANX 23%	NR	
Thomason et al., 2015 [33]	21	12.77 (2)	71.43	*Children’s Trauma* *Assessment Center Screen Checklist.* *Neglect*: 14%; *Emotional abuse*: 10%; *Physical abuse*: 19%; *Domestic violence*: 67%; *Other violence*: 52%; *Multiple separations from parent or caregiver*: 19%	NR	Psychotropic medications, 14%	21	12.32 (2.19)	66.67	NR	Psychotropic medications, 5%	
Fareri et al., 2017 [50]	41	12.33 (2.95)	68	*Previous institutionalized children vs. children raised with biological family from birth*	NR	Psychotropic medications, 22%	47	12.15 (3.54)	57.45	0	0	
Hoffman et al., 2018 [37]	29	14.50 (1.71)	48	*Maltreatment scale* *Neglect*: 72%; *Emotional abuse*: 97%; *Sexual abuse 10%*; *Physical Abuse 14%*; *Domestic violence 59%*	NR	NR	39	14.83 (1.22)	67	NR	NR	
Pang et al., 2021 [17]	40	22.90 (3.30)	55	*CTQ* *Emotional neglect*: 47.5%; *Physical neglect*: 62.5%; *Emotional abuse*: 12.5% *Sexual abuse*: 12.5%; *Physical abuse*: 20%	0	0	50	22.76 (3.46)	60	0	0	
Mattheiss et al., 2022 [38]	23	23.70 (NR)	30	Adapted version of the *Survey of exposure to Community Violence*	0	0	23	22.57 (NR)	57	0	0	
Sokołowski et al. 2022 [39]	51	31.94 (6.21)	59	SCID-I for DSM-IV-TR *Neglect*: 25% *Abuse*: 35%	Different diagnoses: 37%	NR	35	31.66 (6.87)	60	0	0	
Gerin et al. 2023 [51]	40	14.4 (1.6)	47.5%	Maltreatment history based on social services report *Neglect*: 75%; *Emotional abuse*: 97%; *Sexual abuse*: 10%; *Physical abuse*: 15%; *Domestic violence*: 57%	NR	NR	42	14.9 (1.3)	61.9	NR	NR	

*ACEs*: Adverse Childhood Experiences; *NEMESIS*: The Netherlands Mental Health Survey and Incidence Study; *MDD*: Major Depressive Disorder; *ANX*: Anxiety Disorder; *NR*: Not Reported; *CTQ*: Childhood Trauma Questionnaire; *SCID*: The Structured Clinical Interview for DSM; *0*: exclusion criteria include the presence of a mental health diagnosis or the use of psychotropic medications

**Table 2:** Summary of seed-networks of 8 studies included in the meta-analysis

Study	Seed	Salience Network (Participants positive for ACEs = 482; Participants negative for ACEs = 538)	Default Mode Network (Participants positive for ACEs = 514, Participants negative for ACEs = 440)	Central Executive Network (Participants positive for ACEs = 23; Participants negative for ACEs = 23)
Van der Weff et al., 2013 [49]	R amygdala	X		
	L amygdala	X		
	L dACC	X		
	R dACC	X		
	PCC		X	
	L dmPFC		X	
Thomason et al., 2015 [33]	BL amygdala	X		
	CM amygdala	X		
	SF amygdala	X		
Fareri et al., 2017 [50]	Ventral striatum (nucleus accumbens, ventral caudate and ventral putamen)	X		
Hoffman et al., 2018 [37]	R inferior sgACC	X		
	L inferior sgACC	X		
	R superior sgACC	X		
	L superior sgACC	X		
Pang et al., 2021 [17]	R pI	X		
	R IPL		X	
	L IPL		X	
Mattheiss et al., 2022 [38]	R amygdala	X		
	L amygdala	X		
	R DLPFC			X
Sokołowski et al., 2022 [39]	Right precuneus		X	
	L dmPFC		X	
	Right PCC		X	
	Left MTG		X	
	Left angular gyrus		X	
	Right angular gyrus		X	
Gerin et al., 2023 [51]	mPFC		X	
Total seed ROIs		15	11	1

*ACEs*: Adverse Childhood Experiences; *R*: right; *L*: left; *dACC*: dorsal anterior cingulate cortex; *PCC*: posterior cingulate cortex; *dmPFC*: dorso-medial prefrontal cortex; *BL*: basolateral; *CM*: centro-medial; *SF*: superficial; *sgACC*: subgenual anterior cingulate cortex; *pI*: posterior insula; *IPL*: inferior parietal lobule; *DLPFC*: dorsolateral prefrontal cortex; *MTG*: medial temporal gyrus; *mPFC*: prefrontal cortex: medial prefrontal cortex

### Assessment of risk of bias and study quality

A tailored version of the Imaging Methodology Quality Assessment Checklist [44] (Table S1) was used to assess the quality for each study included in the meta-analysis. This tool assesses the quality of studies based on three domains: selection of study groups; methods for image acquisition and analysis; results and conclusions. Each study can be awarded a maximum of one point for each item: the higher the score, the better the quality of the study and the lower the risk of bias. Two reviewers (GMG and GC) independently assessed each study for quality and risk of bias (Table S2) and any potential disagreement was resolved by a third reviewer.

### Seed network parcellation

Each seed from each study was categorized into one of the three brain functional networks (SN, DMN or CEN), following the approach described in a previous meta-analysis [30]. We relied on Menon’s triple-network description [20] as the primary framework, complemented by other parcellations [45, 46]. Assignment was carried out manually, by visually inspecting the reported MNI coordinates (when MNI coordinates were not available, we used the location of seeds as illustrated in figures or atlases, e.g., Talairach) onto these network parcellations. Two independent raters (GMG and GC) performed the classification, and discrepancies were resolved by consensus. All original seed coordinates are listed in Supplementary Table S3, and the final network assignment for each seed is reported in Table 2.

### Meta-analysis and assessment of heterogeneity

The meta-analysis was performed using the Seed-based d Mapping (SDM) software v6.21 (http://www.sdmproject.com/). Each seed was assigned to one of the three networks and was treated as a separate study, with its own corresponding sample and specific peak effects [30]. Therefore, three separate SDM meta-analyses were performed for each of the three networks. Peak coordinates and t values for the effects of the seed belonging to a specific network were entered into SDM. Then, the software created signed maps and effect-sizes maps for each individual seed, by assigning a value to the voxels close to each of the reported coordinates within a map (based on the Talairach Daemon) [47]. Once all the studies had their signed map created, a meta-analytic signed map was calculated by SDM (SDM-Z map).

Finally, in order to evaluate the significance of SDM-Z values at the whole-brain level, a null distribution was created using 1000 subject-based permutations. For each permutation, the maximum statistic across all voxels was recorded. The resulting distribution of maximum statistics was then used to assess the significance of the observed SDM-Z values while effectively correcting for multiple comparisons by controlling the family-wise error rate. Results were then thresholded at a voxel-wise uncorrected significance level of *p* < 0.05. The resulting peak effects were identified and labelled using their MNI coordinates and the Atlas of the Human Brain [48]. To assess heterogeneity between studies, we extracted values from relevant peaks and inspected the corresponding *I*^2^ estimates.

## Results

**Fig. 1:**
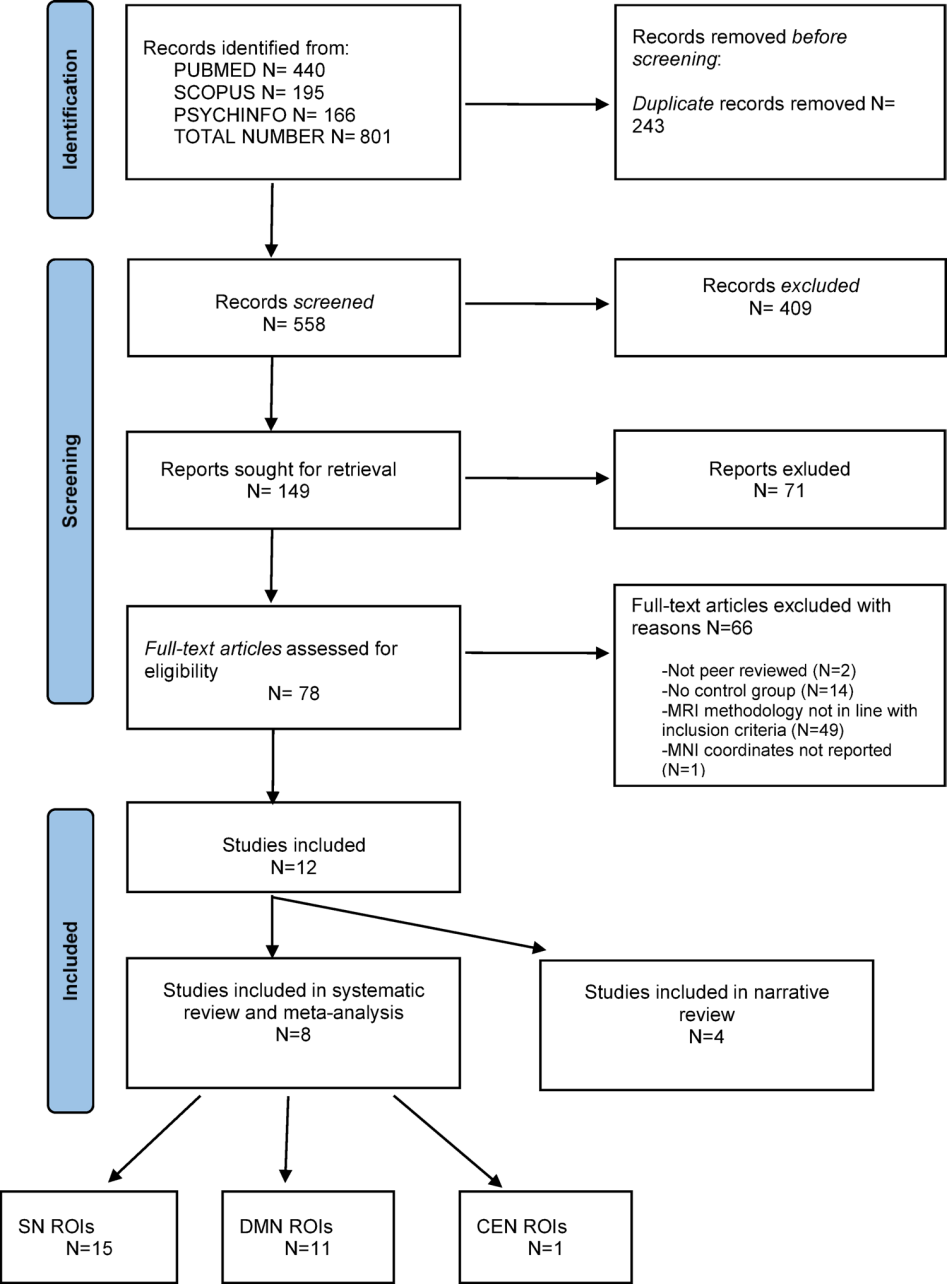
Prisma Flow Diagram. *SN*: Salience Network; *DMN*: Default Mode Network; *CEN*: Central Executive Network; *ROIs*: Regions of Interest.

Of 801 articles identified, 12 could be included in this paper (Fig. 1) [15, 17, 33, 37–39, 49–54]. Of these, eight studies investigated exposure to ACEs in individuals from the general population [17, 33, 38, 39, 49] or in individuals recruited from social care [37, 49–51], while four studies examined the effect of ACEs specifically in individuals with a diagnosis of Major Depressive Disorder (MDD) [15, 52–54].

Given the distinct nature of the populations studied- general population/social care settings versus individuals with a clinical diagnosis of MDD - and the substantial differences in statistical methods (e.g., two-group comparisons in the eight studies conducted in general population/social care settings samples versus three- or four-group comparisons in the studies conducted in MDD), these two groups of studies were therefore analyzed separately. However, studies conducted in MDD samples were few and use different neuroimaging sequences (blood-oxygen level-dependent in three studies and arterial spin labelling in one study) and statistical methods, making it impossible to perform a meta-analysis on their findings [15, 52–54]. We therefore included in the meta-analysis only the eight papers conducted in the general population or social care settings [17, 33, 37–39, 49–51], and provide a narrative review of the results of studies specifically evaluating individuals with a diagnosis of MDD.

### Resting state functional connectivity in general population or social care samples

These eight studies included a total of 289 participants (females = 54%; age 12–39 years) exposed to ACEs and 301 participants not exposed (females = 59%; age 12–37 years). ACEs included emotional and physical neglect, sexual and physical abuse, domestic violence, and separation from parents or caregiver, and were assessed with a variety of instruments (See Table 1).

A diagnosis of mental disorders was an exclusion criterion for both groups (exposed and not exposed to ACEs) in two studies [17, 38], and for the control group in two studies [39, 50]. In contrast, the study by van der Werff and colleagues (2013) included both individuals without a mental health diagnosis, individuals with a diagnosis of anxiety, depression or both, with or without a history of ACEs [49]. In the remaining three studies, details on the presence or absence of a mental disorder were not reported [33, 37, 51]. The demographic and clinical characteristics of participants are presented in Table 1. The MRI methods and main results of resting-state functional connectivity reported in these studies are described in Supplementary Table S3 and in Figure S1.

The network parcellation organised the seed coordinates obtained in each individual study into separate functional networks, resulting in 15 SN seeds, 11 DMN seeds, and 1 CEN seed. Studies that used multiple seeds within the same network were included multiple times in the analysis, each with its corresponding sample size multiplied accordingly. Cluster sizes are given in voxels, which is 2 × 2 × 2mm^3^ isotropic in the MNI152 standard space.

**Fig. 2:**
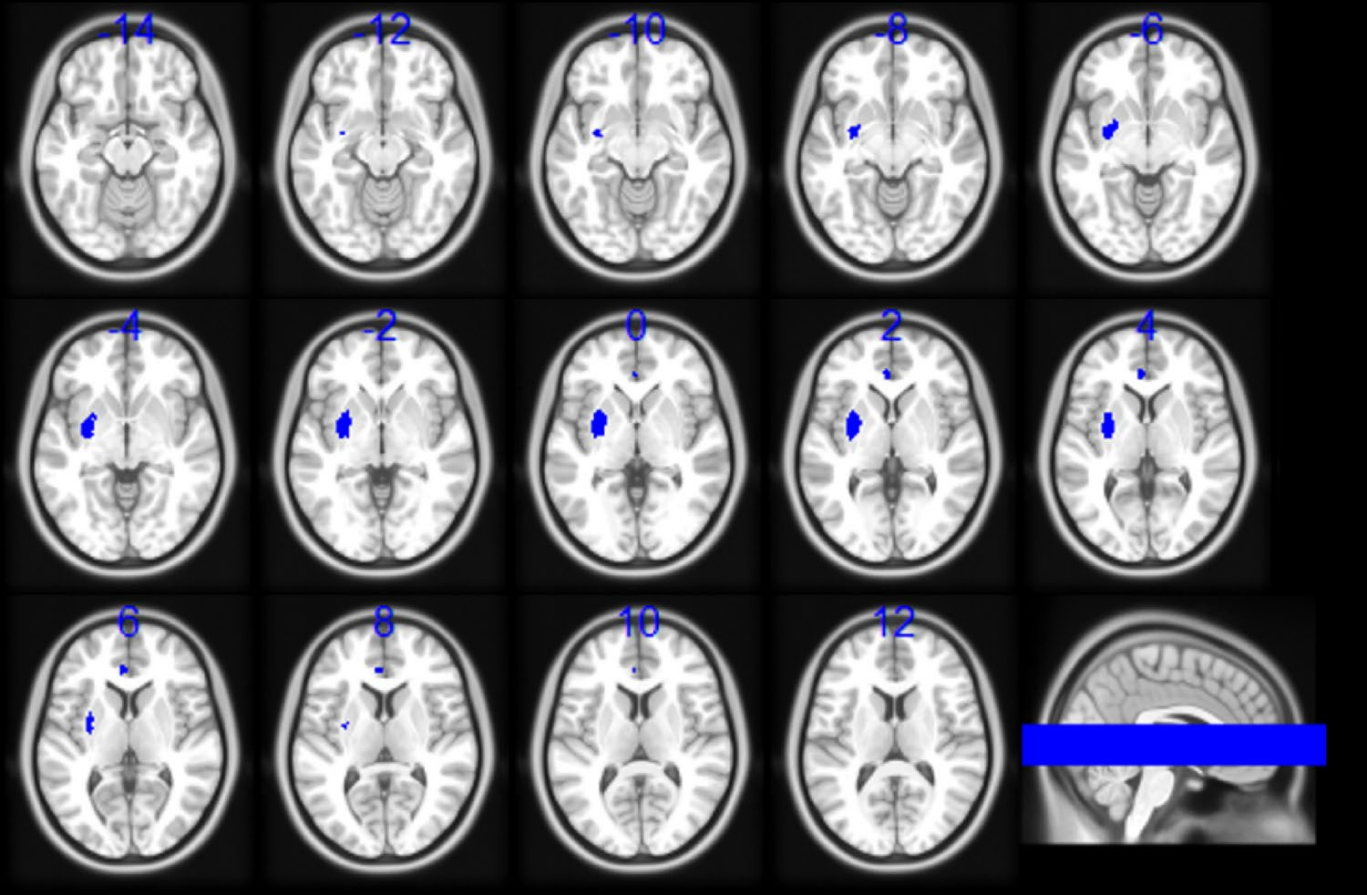
Results of the meta-analysis of the Salience Network (SN). Participants positive for adverse childhood experiences (ACEs), as compared to participants negative for ACEs, showed a pattern of hypoconnectivity (blue colour) between the SN seeds with two clusters, one located in the left lenticular nucleus, putamen and hippocampus, and the other located in the left anterior cingulate, dorsal anterior cingulate cortex and paracingulate gyri

The evaluation of the Salience Network included 482 participants positive for ACEs and 538 participants negative for ACEs, and identified two distinct clusters of hypoconnectivity (Table 3; Fig. 2). The first cluster was located in the left lenticular nucleus, putamen and hippocampus (BA 48) (MNI coordinates: -30, -4, 2; size: 294 voxels; Hedge’s g: -0.198574; I^2^: 17.126091), possibly underlying alterations in goal-directed behavior, emotion regulation, fear conditioning, memory and affective learning processing. The second cluster was centred in the left ACC, dACC (BA 32) and paracingulate gyri (MNI coordinates: -4, 38, 6; size: 38 voxels; Hedge’s g: -0.182; I^2^: 24.88), which could underlie disruptions in emotion regulation, as well as the difficulties in processing social information previously associated with ACEs.

**Table 3:** Results of meta-analyses for the salience and default mode networks

Network and Seeds	Between-groups comparison	Peak effect	Subpeak effect	Coordinates			SDM-Z	P-value
				x	y	z		
SN: Amygdala; dACC; sgACC; ventral; pI	Participants positive for ACEs < Participants negative for ACEs	Left lenticular nucleus, putamen, hippocampus 294 voxels		-30	-4	2	-2.420	0.007759690
			Left lenticular nucleus, putamen	-28	2	2	-2.301	0.010685503
			“undefined”, BA 48	-32	2	2	-2.236	0.012672126
			left inferior network, inferior fronto-occipital fasciculus	-32	-4	-10	-1.992	0.023192346
		Left anterior cingulate/paracingulate gyri, dACC 38 voxels		-4	38	6	-1.970	0.024410248
DMN: PCC; dMPFC; IPL; precuneus; MTG; angular gyrus	Participants positive for ACEs < Participants negative for ACEs	Left middle frontal gyrus, DLPFC 164 voxels		-40	38	24	-2.500	0.006212354
			Left middle frontal gyrus, BA 46	-40	38	28	-2.193	0.014154494

*ACEs*: Adverse Childhood Experiences; *DMN*: Default Mode Network; *SN*: Salience Network; *dACC*: dorsal anterior cingulate cortex; *sgACC*: subgenual anterior cingulate cortex; *pI*: posterior insula; *PCC*: posterior cingulate cortex; *dmPFC*: dorso-medial prefrontal cortex; *IPL*: inferior parietal lobule; *MTG*: medial temporal gyrus; *DLPFC*: dorsolateral prefrontal cortex

**Fig. 3:**
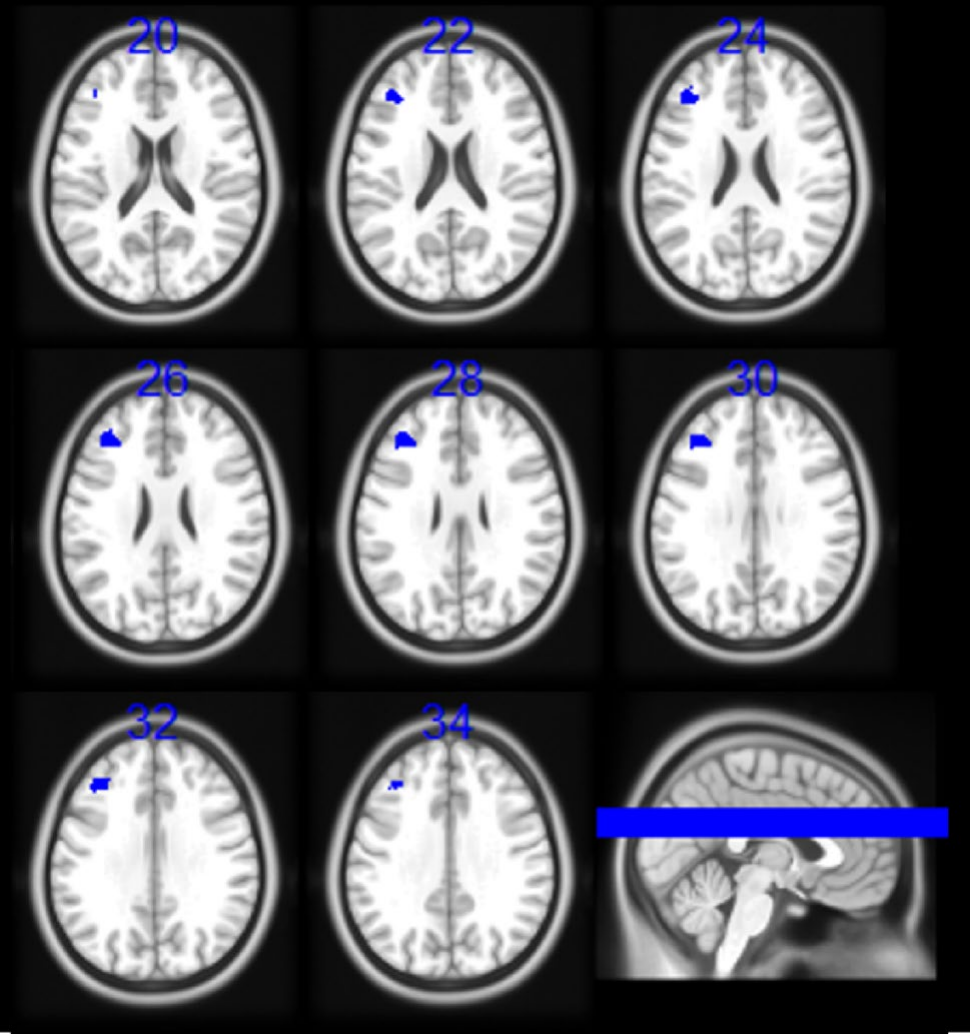
Results of the meta-analysis of the Default Mode Network (DMN). Participants positive for adverse childhood experiences (ACEs), as compared to participants negative for ACEs, showed a pattern of hypoconnectivity (blue colour) between the DMN seeds with left middle frontal gyrus and dorso-lateral prefrontal cortex

The evaluation of the DMN included 514 participants positive for ACEs and 440 participants negative for ACEs. There was a cluster of hypoconnectivity in individuals positive for ACEs in the left middle frontal gyrus and DLPFC (BA 46) (MNI coordinates: -40, 38, 24; size: 164 voxels; Hedge’s g: -0.0234; I^2^: 23.14) (Table 3; Fig. 3). This finding points to an altered functional integration of regions involved in higher-order cognitive functions, such as decision making and cognitive control, impairments which have been reported in individuals exposed to adverse childhood experiences [16].

Finally, as only one study reported a whole-brain map from a seed corresponding to the CEN network, it was not possible to conduct a meta-analysis of evidence relating to this network [38]. This study found that exposure to ACEs was significantly associated with reduced connectivity between the right DLPFC and the left middle frontal gyrus, right superior frontal gyrus, left inferior parietal sulcus, right anterior cingulate cortex, left caudate and right cerebellum [38].

### Resting state functional connectivity in studies of participants with major depressive disorder

The four studies that specifically included individuals with MDD included 219 participants with MDD (females = 52%; age 22–35 years) exposed to ACEs, 119 participants with MDD not exposed to ACEs (females = 60%; age 22–40 years), 82 controls (HC) previously exposed to ACEs (females = 51%, age 20–41 years) and 145 controls not exposed to ACEs (females = 52%, age 20–34 years). All studies assessed exposure to ACEs using the Childhood Trauma Questionnaire (CTQ), evaluating emotional and physical neglect, as well as emotional, sexual and physical abuse. Table 4 summarizes the demographic and clinical characteristics of participants included in these studies.

**Table 4:** Demographic and clinical characteristics of the 4 studies including participants with MDD

Exposed to ACEs: -Participants positive for MDD and ACEs (*N* = 219) -Participants negative for MDD and positive for ACEs (*N* = 82)							Unexposed to ACEs: -Participants positive for MDD and negative for ACEs (*N* = 119) -Participants negative for MDD and for ACEs- (*N* = 145)				
Study	N	Mean age (SD)	Female (%)	Adversities tools	Psychiatric Comorbidities	Psychiatric Medications	N	Mean age (SD)	Female (%)	Psychiatric Comorbidities	Psychiatric Medications
Wang et al., 2022 [15]	91	22.41 (4.01)	56.04	CTQ	100% MDD (other comorbidities NR)	Psychotropic medications, 32%	32	22.63 (4.03)	65.63	100% MDD (other comorbidities NR)	Psychotropic medications, 31%
	33	20.73 (3.42)	51.52		0	0	46	22.39 (4.11)	52.17	0	0
Fan et al., 2023 [52]	42	28.83 (5.91)	54.76	CTQ	100% MDD (First episode)	0	31	22.97 (5.85)	74.19	100% MDD (First episode)	0
	30	20.47 (2.71)	53.33		0	0	34	20.82 (2.73)	50.00	0	0
Schirmer et al.,2022 [53]	22	31.86 (10.07)	50	CTQ	100% MDD, of which 63.64% in comorbidity with ANX	Antidepressant 50%	23	39.96 (13.96)	43.48	100% MDD of which 47.83% in comorbidity with ANX	Antidepressant 65.22%
							23	33.78 (11.72)	43.48	0	0
Liu et al., 2024 [54]	55	35.62 (12.38)	42	CTQ	100% MDD (other comorbidities NR)	NR	34	40.53 (13.79)	53	100% MDD (other comorbidities NR)	NR
	19	41.37 (12.83)	47		0	0	42	34.52 (11.4)	57	0	0

*MDD*: Major Depressive Disorder; *ACEs*: Adverse Childhood Experiences; *NR*: Not Reported; *CTQ*: Childhood Trauma Questionnaire; *ANX*: Anxiety Disorder; *0*: exclusion criteria include the presence of a mental health diagnosis or the use of psychotropic medications

As mentioned above, due to the paucity of studies and the large heterogeneity of neuroimaging sequences (blood-oxygen level-dependent in three studies and arterial spin labelling in one study) as well as of statistical methods used, we could not perform a meta-analysis of the findings of these studies [15, 52–54], and only provide below a narrative review of their findings (see also Table S4). Results from these studies were not always consistent. For example, Fan and colleagues [52] reported a pattern of decreased rs-FC of right amygdala-precuneus, in both individuals with depression and healthy controls previously exposed to ACEs, but no significant interaction of diagnosis x ACEs exposure. In addition, the right amygdala–precuneus connectivity was found to mediate the association between ACEs and trait anhedonia in participants with MDD [52]. This suggests that aberrations in the amygdala–precuneus pathway in individuals positive for ACEs may be associated with a reduced capacity to experience pleasure, possibly due to deficits in emotion regulation through impairment in attention, which may contribute to the development of low mood following ACEs exposure [52].

Two studies found a significant effect of diagnosis x ACEs exposure, with MDD individuals positive for ACEs showing a pattern of hypoconnectivity in various neural pathways compared to those with MDD but negative for ACEs [15, 54]. Specifically, Liu et al. (2024) reported hypoconnectivity in the left orbitofrontal cortex (OFC)-right DLPFC, right OFC-right ventromedial PFC, and left lateral PFC-left visual cortex [54]. Wang et al. (2022) reported hypoconnectivity in the left inferior temporal gyrus-precuneus/PCC/middle temporal gyrus/left medial OFC/bilateral medial PFC/ACC [15]. Interestingly, the connectivity pattern was reversed in controls positive for ACEs, who showed a pattern of neural hyperconnectivity compared to controls negative for ACEs [15, 54]. It is possible that a higher rs-FC in healthy controls exposed to ACEs reflects an adaptive response, through alternative developmental pathways that could promote reproduction and survival. Interestingly, in the study by Wang and colleagues (2022), aberrations in the rs-FC of the left inferior temporal gyrus- precuneus/PCC and left medial OFC mediated the relationship between ACEs and depression in participants with MDD, suggesting that the neural mechanisms of MDD following ACEs exposure may be related to a dysfunction of the Default Mode Network [15].

Finally, Schirmer and colleagues [53] reported that participants with MDD (exposed and not exposed to ACEs) showed a pattern of hypoconnectivity when compared to healthy controls. In particular, those positive for MDD and ACEs showed decreased connectivity between right PCC-left fusiform gyrus, right dorsal anterior insula-left ACC, left dorsal anterior insula-left superior frontal gyrus, and those positive for MDD but negative for ACEs exposure showed decreased connectivity between the right PCC-right precuneus, right dorsal anterior insula-right superior frontal gyrus.

## Discussion

To our knowledge this is the first meta-analysis of studies that evaluated any type of adverse childhood experiences and resting state functional connectivity across multiple brain regions, using a seed-to-whole brain approach, which can specifically uncover relationships or brain patterns that may not emerge with a priori ROI-to-ROI approaches. Our main finding is that individuals exposed to early adversity, compared to those not exposed to these experiences, consistently show decreased connectivity of the Salience and Default Mode Networks. Specifically, we found hypoconnectivity between the Salience Network and the left lenticular nucleus (including the putamen), the hippocampus, the left anterior cingulate cortex and paracingulate gyri, as well as between the DMN and the middle frontal gyrus and the dorsolateral prefrontal cortex. Together, these results suggest that adverse childhood experiences are associated with a pattern of hypoconnectivity within and between large-scale networks. Such alterations seem consistent with models suggesting accelerated maturation or premature disruption of neural systems following early adversity [35, 55]. Given the established roles of the DMN, SN, and CEN in self-referential processing, emotion regulation, and executive control [20, 27, 28], reduced connectivity within and between these networks could contribute to difficulties in these domains. While this interpretation remains tentative, it may offer insights into potential neurobiological pathways through which early adversity increases long-term vulnerability to mental health problems.

### Salience network

Participants exposed to early adversity showed decreased connectivity between regions of the Salience Network, such as the amygdala and insula, and the dorsolateral part of the putamen. The amygdala and the putamen are part of a functional network that also includes the prefrontal cortex, the ventral medial pallidum, and the thalamus [56]. This functional network is thought to be associated with goal-directed behavior and the ability to suppress negative feelings, which are often impaired in individuals exposed to early adversity [49]. Reduced SN–putamen connectivity may therefore indicate alterations in developmental trajectories of this circuit, potentially contributing to difficulties in affect regulation and increased vulnerability to psychopathology in individuals exposed to early adversity [55]. The observation that putamen–insula coupling decreases across development further supports the interpretation that these alterations could reflect accelerated maturation of salience-related pathways [55].

We also found reduced connectivity within regions of the Salience Network, such as the amygdala, insula, anterior cingulate cortex, and hippocampus. Amygdala-hippocampus connectivity has been implicated in fear expression and social behaviors, and reduced coupling in this circuit may contribute to an increased risk of anxiety symptoms and impaired social behaviors, in line with previous findings [57]. Furthermore, the hippocampus has strong connectivity with the subgenual anterior cingulate cortex, a pathway thought to subtend memory and affective learning processes, including the modulation of reinforcement-based learning and fear conditioning. Dysconnectivity in the subgenual anterior cingulate cortex-hippocampus pathway, following the exposure to early adversity, might therefore underlie poor integration of novel information into long-term memory, as well as aberrant fear responses, which can lead to the development of anxiety symptoms [29, 34].

Previous literature on the relationship between early adversity and Salience Network-putamen connectivity is limited. Still, our finding of reduced connectivity between the SN and putamen is consistent with two previous studies that used different methods. For example, Van Der Werff and colleagues (2013), which has been included in the present meta-analysis, using a seed-to-whole brain approach, reported reduced connectivity between the amygdala and putamen in adults exposed to early adversity compared to those not exposed [49]. Similarly, Boecker and colleagues (2014) also found decreased putamen activation in relation to early adversity, evaluating task-based activation [58]. Previous research on SN connectivity with the hippocampus and anterior cingulate cortex shows inconsistent results. Indeed, studies have reported both increased amygdala-hippocampus coupling during emotional face processing or olfactory processing tasks, and decreased coupling during fear conditioning tasks or resting-state conditions [42]. Finally, both increased and decreased SN-ACC coupling have been reported in the literature [22, 29, 34, 35, 38, 59, 60]. Decreased insula-dACC coupling, in particular, has been associated with various outcomes, including a reduced ability to filter goal-irrelevant affective information [22], reduced sustained attention and academic performance, hyperactivity symptoms [60] and social problems [50].

### Default mode network

We found that participants exposed to early adversity showed reduced connectivity within a cluster that included the left middle frontal gyrus and the dorsolateral prefrontal cortex. This pattern may reflect disruptions in the interaction between the Default Mode Network and the Central Executive Network, potentially contributing to difficulties in decision-making processes. Prior studies have implicated connectivity between DMN hubs, particularly in the inferior parietal lobule and frontal regions, in attention control and decision-making processes [61]. These functions have been reported as impaired in individuals exposed to early adversity [62], suggesting that the DMN hypoconnectivity we observed could represent a neural correlate of these difficulties.

Our finding of hypoconnectivity between DMN and frontal regions is consistent with previous fMRI studies, which have observed similar hypoconnectivity associated with ACEs both in resting state conditions [17, 38] and during a sustained attention task [63]. Furthermore, robust evidence supports an association between smaller grey matter volume in frontal regions, including the dorsolateral prefrontal cortex, and a history of early adversity [8, 62, 64], which might be reflected also in a reduction of functional connectivity. Of note, prior research has shown that aberrant functional connectivity between Default Mode Network hubs and frontal regions is associated with negative outcomes such as cyclothymic temperament, attentional impulsivity [17] and interpersonal problem-solving abilities [51], highlighting the clinical significance of these neural mechanisms in the context of early life stress.

Overall, the findings of this meta-analysis indicate that exposure to adverse childhood experience is associated with aberrations in the functional integration between the Salience and the Default Mode Networks, as well as with a disrupted switching between the Default Mode and the Central Executive Networks. These aberrations have been proposed to represent a core pathophysiological mechanism underlying maladaptive outcomes, including increased risk for developing anxiety, depression and psychoses [20]. This disrupted functional connectivity seems to mostly affect brain regions with dense expression of glucocorticoid receptors, such as the prefrontal cortex, anterior cingulate cortex, hippocampus, amygdala and insula [15–17], which are more susceptible to the effects of prolonged or excessive activity of the hypothalamus-pituitary-adrenal axis, following exposure to early adversity. This may alter the typical course of brain development, leading to alterations in synaptic plasticity, neurotransmitter systems, structural integrity, as well as functional connectivity within and between key neural networks. These alterations may contribute to the emergence of problems in social behavior, as well as deficits in emotional, affective and cognitive processes [17, 50, 51]. Finally, altered connectivity between brain regions involved in emotional processing and higher-order cognitive functions may impair adaptive coping strategies and decision-making processes, further exacerbating the risk for psychopathology in vulnerable individuals.

### Strengths and limitations

The main strengths of our meta-analysis are the inclusion of any type of adverse childhood experiences and the use of a seed to whole-brain approach, which can uncover relationships or brain patterns that may not emerge with seed-to-ROI approaches. Our methods also benefit from strict inclusion criteria, which ensured that only high-quality studies were included, enhancing the reliability and validity of findings. However, some limitations should also be acknowledged.

First, there was significant heterogeneity in the studies included, arising from differences in the conceptualization of adverse childhood experiences, in the timing and proximity of these experiences, the characteristics of study participants, the methods used for MRI data processing, and the functional connectivity models employed.

Thus, further research in this area should investigate early adversity in more homogeneous populations, while ensuring that the study conditions authentically reflect the trauma exposure. This includes considering factors such as a higher prevalence of anxiety and/or depressive symptoms commonly associated with adverse childhood experiences.

Different instruments were used to assess ACEs, and not all studies reported details of psychiatric comorbidities. This variability precluded formal subgroup or sensitivity analyses to examine differential effects of specific ACEs or severity levels. Moreover, the potential influence of psychiatric comorbidities on resting-state functional connectivity could not be systematically evaluated due to inconsistent reporting across studies.

A further limitation concerns the timing of ACEs assessment relative to MRI acquisition. Except for one study [33], no study specified the time interval between ACEs exposure and the MRI acquisition. This lack of information makes the interpretation of resting-state connectivity patterns difficult, as variability in the interval between ACEs assessment and neuroimaging acquisition could introduce biases such as recall errors or differences in temporal proximity to the exposure.

Future studies should systematically report the timing between ACEs assessment and MRI acquisition, as well as provide a detailed characterization of participants, e.g., psychiatric comorbidities, since both aspects are crucial for enhancing the interpretability and reproducibility of findings.

An additional limitation is that studies used multiple seeds within a specific network were included more than once in the same analysis. This could have potentially led to a bias in the results. Our meta-analysis also relied on coordinate-based analyses using peak activations, rather than a meta-analysis based on statistics estimated across entire brain maps. Brain maps, as opposed to peak coordinates, naturally provide a much more comprehensive understanding of neural correlates, yet they have seldom availability unlike peak coordinates. Finally, caution should be used about the assumption that resting-state-functional connectivity of a collection of seeds accurately reflects connectivity patterns of larger brain networks. Nonetheless, the definition of these brain networks aligns with previous research, where these were identified based on temporal correlations among the time-series data of specific brain regions [27, 30, 45].

## Conclusions

The results of this meta-analysis provide evidence on the presence of a pattern of hypoconnectivity within the SN, between SN, integrative hubs and DMN, as well as between DMN and CEN in individuals with a history of adversity. As such, aberrations in the functional integration between the SN and the DMN, as well as disrupted switching between DMN and CEN, could represent the neurobiological substrate of maladaptive outcomes following ACEs exposure.

Understanding the complex pathophysiological mechanisms associated with ACEs is crucial for elucidating the neurobiological changes that contribute to disrupting developmental trajectories. Improving our insight into these mechanisms represents a priority in current research efforts, with profound implications for informing targeted interventions and reducing the long-term effects of early adversity on brain development and behavior.

## Supplementary Information

Below is the link to the electronic supplementary material.

Supplementary Material 1
