# Supplementary material for: Adverse childhood experiences and resting state functional connectivity of the triple brain network: a meta-analysis
Source: Eur Arch Psychiatry Clin Neurosci. 2026 Feb 12;276(4):1449–63. doi: 10.1007/s00406-026-02204-2 (PMC13233943; doi:10.1007/s00406-026-02204-2)
Supplement: Supplementary file 1 — Supplementary Material 1 [file 406_2026_2204_MOESM1_ESM.docx]

Supplementary Material for

**Adverse childhood experiences and resting state functional connectivity**

**of the triple brain network: a meta-analysis**

Giulia M. Giordano^1,2*^, Giulia Cattarinussi^2^, Andrew Lawrence^2^, Nare Amasi-Hartoonian^2,3^, Svenja Kretzer^2,4^, Xuemei Ma^2^, Rebecca Pollard^2^, Corentin Vallée^2^, Armida Mucci^1^, Silvana Galderisi^1^, Paola Dazzan^2,3^

^1^Department of Mental and Physical Health and Preventive Medicine, School of Medicine, University of Campania Luigi Vanvitelli, Largo Madonna delle Grazie 1, 80135 Naples, Italy

^2^Department of Psychological Medicine, Institute of Psychiatry, Psychology and Neuroscience, King’s College London, 16 De Crespigny Park, London SE5 8AF, London, UK

^3^NIHR Maudsley Biomedical Research Centre at South London and Maudsley NHS Foundation Trust, London, UK

^4^Singapore Institute for Clinical Sciences, Agency for Science, Technology & Research (A∗STAR) Singapore, Republic of Singapore

*Corresponding author:

Giulia Maria Giordano, Department of Mental and Physical Health and Preventive Medicine, School of Medicine, University of Campania Luigi Vanvitelli, Largo Madonna delle Grazie 1, 80135 Naples, Italy

giuliamaria.giordano@unicampania.it

**INDEX**

Supplementary Table S1……………………………………………………………………………...3

Supplementary Table S2……………………………………………………………………………...4

Supplementary Table S3……………………………………………………………………………...5

Supplementary Figure S1……………………………………………………………………………..8

Supplementary Table S4……………………………………………………………………………... 9

References…………………………………………………………………………………………… 10

**Supplementary Table S1** Adapted Imaging Methodology Quality Assessment Checklist

|  | **Category 1: Participants** | **Score** (0/0.5/1) |
| --- | --- | --- |
| 1 | People with a history of ACEs exposure were included. Specific criteria were used in the assessment of exposure. Demographic data were reported |  |
| 2 | People without a history of ACEs exposure were included and matched to the case cohort. Demographic data were reported |  |
| 3 | Key variables (e.g., age, gender, handedness, height or total brain measures) were checked, either by stratification or statistically |  |
| 4 | Sample size per group > 10 |  |
|  | **Category 2: Methods for image acquisition and analysis** |  |
| 5 | All neuroanatomic measurements were taken without considering group assignment or subject identity |  |
| 6 | Magnet strength > 1T |  |
| 7 | The imaging technique used was clearly described so that it could be reproduced |  |
| 8 | Measurements were clearly described so that they could be reproduced |  |
|  | **Category 3: Results and conclusions** |  |
| 9 | Statistical parameters for significant, and important non-significant, differences were provided |  |
| 10 | Conclusions were consistent with the results obtained and the limitations were discussed |  |
|  | **TOTAL** | /10 |

The score for each item ranged from 0 to 1, where 0.5 was assigned when criteria were partially met. This qualitative rating aimed to describe the methodological limitation of published studies to help the reader in weighting the importance of the findings of a study.

**ACEs:** Adverse Childhood Experiences

Modified from: Shepherd et al., 2012 (1)

**Supplementary Table S2** Assessment of quality and risk of bias

| **Study** | **Quality** | **Risk of bias** | | | |
| --- | --- | --- | --- | --- | --- |
|  |  | Participants | Methods | Results and Conclusions | Overall |
| *Van der Weff et al., 2013 (2)* | 8 | - | + | + | + |
| *Thomason et al., 2015 (3)* | 7.5 | + | - | - | - |
| *Fareri et al., 2017 (4)* | 8 | - | + | + | + |
| *Hoffman et al., 2018 (5)* | 9 | + | + | + | + |
| *Pang et al., 2021 (6)* | 9.5 | + | + | + | + |
| *Mattheiss et al., 2022 (7)* | 8 | - | + | + | + |
| Sokołowski *et al., 2022(8)* | 9.5 | + | + | + | + |
| *Gerin et al., 2023 (9)* | 9 | + | + | + | + |
| *Wang et al., 2022(10)* | 9 | + | + | + | + |
| *Fan et al., 2023 (11)* | 9 | + | + | + | + |
| *Schirmer et al. 2023(12)* | 8 | - | + | + | + |
| *Liu et al. 2024 (13)* | 9 | + | + | + | + |

+ = Low risk of bias; - = Medium risk of bias; x = High risk of bias

**Supplementary Table S3** Resting-state functional connectivity reported in the eight studies including individuals from the general population or individuals recruited from social care settings

| **Study** | **Eyes open/closed;**  **MRI processing software;**  **Magnetic Field; Functional connectivity model** | **Seeds**  (coordinates/atlas) | **Main Results***  Participants positive for ACEs vs participants negative for ACEs (peak MNI coordinates) |
| --- | --- | --- | --- |
| *Van der Weff et al., 2013 (2)* | NR;  FSL;  3T;  Linear regression | R AMY (26,-2,-18);  L AMY (-20,-6,-16);  R dACC (6,18,28);  L dACC (-6,18,28);  PCC (-2,-36,36);  L dmPFC (-11,23,40) | **Participants positive for ACEs < Participants negative for ACEs**  R AMY-hippocampus (-34,-24,-6)  R AMY-insula (-36,-8,-6)  R AMY-putamen (-34,-12,-4)  R AMY-temporal-occipital cortex (-40,-44,-12)  L dACC-paracingulate (-8,-38,0)  L dACC-frontal medial cortex (-12,44,-16) |
| *Thomason et al., 2015(3)* | Closed;  CONN;  3T;  Semipartial correlation | CM, BL and SF AMY (SPM Anatomy toolbox) | **Participants positive for ACEs < Participants negative for ACEs**  CM AMY-sensorimotor (-4,-28,74; 4,-20,50)  CM AMY-PCC (-20,-52,24)  BL AMY-STG (-54,16,-8)  BL AMY-precuneus (18,-76,36)  BL AMY-putamen (30,-14,14)  SF AMY-dACC (2,38,12)  SF AMY-midbrain (0,-30,-6)  **Participants positive for ACEs > Participants negative for ACEs**  CM AMY- precentral gyrus (-46,-2,36)  CM AMY- precuneus (20,-76,48)  CM AMY- OFC (-16,26,-28)  CM AMY- sgACC (8,18,-8)  CM AMY- hippocampus (16,-6,-24)  CM AMY- lentiform (-10,2,-4)  BL AMY- MFG (-44,8,46)  BL AMY- SFG (-26,14,54)  BL AMY- OFC (-26,60,-6)  BL AMY- superior parietal (-6,-72,58)  SF AMY- MFG (42,14,38)  SF AMY- sensorimotor (-2,-28,72)  SF AMY- SFG (-6,50,34)  SF AMY- ITG (-46,-2,-40)  SF AMY- MTG (-54,-28,-4)  SF AMY- MOG (30,-96,4)  SF AMY- insula (-46,10,-8) |
| *Fareri et al., 2017 (4)* | Closed;  AFNI;  3T;  linear regression | VS (FSL atlas) | **Participants positive for ACEs > Participants negative for ACEs**  VS- R ACC (15,42,3)  VS- L ACC (12,48,7)  VS- L SFG (-5,55,31)  VS- R MFG (15,69,8)  VS- R subcallosal gyrus (18,27,-13)  VS- L caudate (-5,19,15) |
| *Hoffman et al., 2018 (5)* | NR;  SPM8;  1.5T;  Z Fisher correlation  coefficient | R superior sgACC (5,25,10);  L superior sgACC (-5,-25,-10);  R inferior sgACC (5,34,4);  L inferior sgACC (-5,-34,-4) | **Participants positive for ACEs < Participants negative for ACEs**  R superior sgACC-R SMG (60,-42,30)  R superior sgACC-R DLPFC (33,30,36)  R superior sgACC-cuneus (0,-87,21) |
| *Pang et al., 2021 (6)* | Closed;  CONN;  3T;  Pearson correlation coefficient | R pI (42,-12,-6);  R IPL (58,-40,38);  L IPL (-54,-48,44) | **Participants positive for ACEs < Participants negative for ACEs**  R pI-R daI (48,9,-6)  R pI-L STG (-66,-30,18)  R IPL-L STG (-42,-15,-3)  R IPL- L MFG (-36,30,27)  L IPL- R IPL (57,-45,39)  L IPL- L MFG (-33,39,24)  L IPL- dACC (-12,30,30)  **Participants positive for ACEs > Participants negative for ACEs**  R IPL- cuneus (-3,-69,18)  L IPL- L SFG (-21,36,54) |
| *Mattheiss et al., 2022 (7)* | NR;  AFNI;  3T;  linear regression | Bilateral AMY (Talairach atlas);  R DLPFC (44,32,31) | **Participants positive for ACEs < Participants negative for ACEs**  R AMY- R temporal pole (27,-13,-26)  R AMY- L ITG (-60,-18,-25)  R AMY- L parahipp gyrus (-28,2,-34)  R AMY- ACC (2,34,18)  R AMY- L putamen (-21,-7,-9)  R DLPFC- L MFG (-15,44,16)  R DLPFC- L IPS (-42,-34,53)  R DLPFC- R SFG (24,53,8)  R DLPFC- R ACC (14,18,25)  R DLPFC- L caudate (-20,23,7)  R DLPFC- R cerebellum (34,-53,37) |
| Sokołowski *et al., 2022 (8)* | Open;  CONN;  3T;  Z Fisher correlation  coefficient | R precuneus (4,-52,28);  L dmPFC (-2,56,40);  R PCC (2,-20,36);  L MTG (-62,-14,-10);  R angular gyrus (50,-5,34);  L angular gyrus (-50,-60,26) | **Participants positive for ACEs < Participants negative for ACEs**  L MTG- L IFG (-52,12,-2)  L dmPFC-R planum temp (66,-34,18)  L dmPFC- L IFG (-50,16,28)  **Participants positive for ACEs > Participants negative for ACEs**  R angular gyr-L cerebellum (-18,-50,-28) |
| *Gerin et al., 2023 (9)* | Open;  CONN;  1.5T;  Z Fisher correlation  coefficient | mPFC (1,55,-3) | **Participants positive for ACEs > Participants negative for ACEs**  mPFC- PCC (12,-51,3) |

**ACC**: anterior cingulate cortex; **ACEs**: Adverse Childhood Experiences; **AFNI**: Analysis of Functional NeuroImages; **AMY**: amygdala; **BL**: basolateral; **CM**: centro-medial; **CONN**: functional connectivity toolbox; **DLPFC**: dorsolateral prefrontal cortex; **dACC**: dorsal anterior cingulate cortex; **daI**: dorsal anterior insula; **DPABI**: Data Processing & Analysis for Brain Imaging; **DPARSF**: Data Processing Assistant for Resting-State fMRI; **IFG**: inferior frontal gyrus; **IPL**: inferior parietal lobule; **ITG**: inferior temporal gyrus; **L**: left; **MFG**: middle frontal gyrus; **MNI**: Montreal Neurological Institute; **MOG**: medial occipital gyrus; **MRI**: magnetic resonance imaging; **MTG**: medial temporal gyrus; **mPFC**: prefrontal cortex: medial prefrontal cortex; **NR**: not reported; **OFC**: orbitofrontal cortex; **PCC**: posterior cingulate cortex; **pI**: posterior insula; **R**: right; **SF**: superficial; **SFG**: superior frontal gyrus; **sgACC**: subgenual anterior cingulate cortex; **SMG**: superior middle gyrus; **SPM**: Statistical Parametric Mapping; **STG**: superior temporal gyrus; **VS**: ventral striatum.

***** Main results are reported as differences between groups on resting-state functional connectivity

**Supplementary Figure S1**


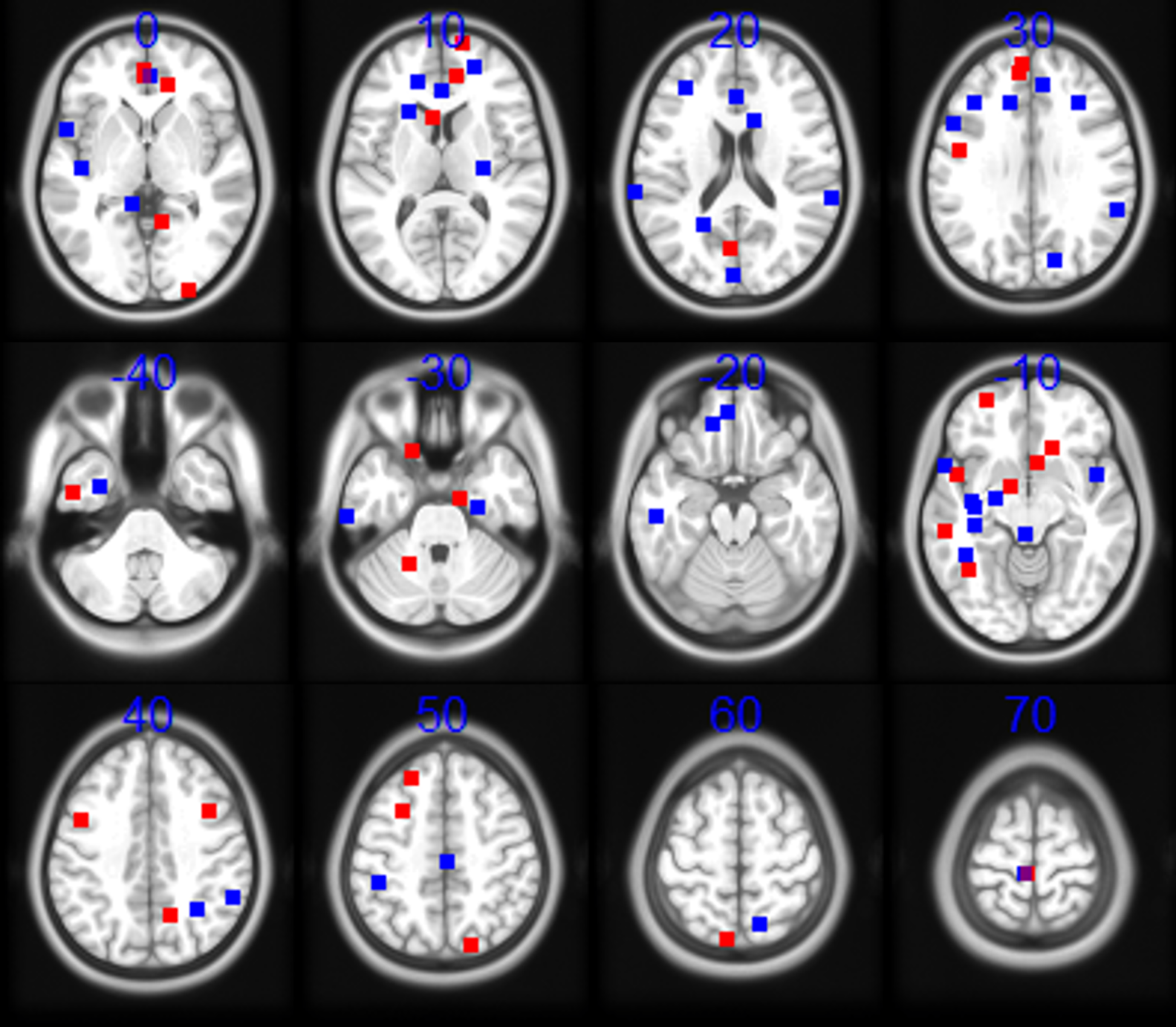


Brain map showing peak MNI coordinates of differences between participants exposed to Adverse Childhood Experiences (ACEs) and those not exposed, as reported by studies included. The z-axis coordinates of the slices are shown on top in blue. Peak-voxels are inflated into 5x5x5 cube of voxels centred on the peak coordinates for easier representation. Red corresponds to positive association (i.e. the presence of ACEs associated with increased connectivity at the location); blue corresponds to negative association.

**Supplementary Table S4** Resting-state functional connectivity reported in the four studies including participants with Major Depressive Disorder

| **Study** | **Eyes open/closed;**  **MRI processing software;**  **Magnetic field; Functional connectivity model** | **Seeds**  (coordinates/atlas) | **Main Results***  (peak MNI coordinates) |
| --- | --- | --- | --- |
| *Wang et al., 2022 (10)* | Closed;  DPARSF;  3T;  Pearson correlation coefficient | L ITG (-39,-18,-30) | **MDD+ ACEs+ < MDD+ ACEs-**  **HC ACEs+ > HC ACEs-**  L ITG-precuneus (9, -51, 18)  L ITG- L MTG (-51,-18,-21)  L ITG- L OFC (-3,51,-15)  L ITG- bilateral mPFC (9,42,30) |
| *Fan et al., 2023 (11)* | NR;  DPARSF;  3T;  NR | R hippocampus (29,-25,-11);  L hippocampus (-31,-25,-11)  R AMY (21,-6,-20);  L AMY (-23,-6,-20) | **MDD+ ACEs+ HC ACEs+ < MDD+ ACEs- HC ACEs**-  R AMY- precuneus (15,-57,42) |
| *Schirmer et al., 2023 (12)* | Closed;  DPARSF;  3T;  Interregional correlation | R PCC;  dmPFC;  AMY;  dACC;  bilateral daI  (NR) | **MDD+ ACEs+ < HC**  RPCC- L fusiform gyrus (-39,-54,-12)  R daI-L ACC (0,48,6)  L daI- L SFG (0,51,6)  **MDD+ ACEs- < HC**  RPCC- R precuneus (15,-63,60)  R daI-R SFG (3,48,-3) |
| *Liu et al., 2024 (13)* | Closed;  DPABI;  3T;  Pearson correlation coefficient | Bilateral OFC;  Lateral PFC  (NR) | **MDD+ ACEs+ < MDD+ ACEs-**  **HC ACEs+ > HC ACEs-**  L OFC- R DLPFC  R OFC- R vmPFC  Lateral PFC- L visual cortex  (NR) |

**ACC**: anterior cingulate cortex; **ACEs**: Adverse Childhood Experiences; **AMY**: amygdala; **DLPFC**: dorsolateral prefrontal cortex; **dACC**: dorsal anterior cingulate cortex; **daI**: dorsal anterior insula; **DPABI**: Data Processing & Analysis for Resting-State fMRI; **DPARSF**: Data Processing Assistant for Resting-State fMRI; **HC**: healthy controls; **HC ACEs+**: healthy controls exposed to ACEs; **HC ACEs-**: healthy controls unexposed to ACEs; **ITG**: inferior temporal gyrus; **L**: left; **MDD**: Major Depressive Disorder; **MDD+ACEs+**: subjects with MDD exposed to ACEs; **MDD+ACEs-**: subjects with MDD unexposed to ACEs; **MNI**: Montreal Neurological Institute; **MRI**: magnetic resonance imaging; **MTG**: medial temporal gyrus; **mPFC**: medial prefrontal cortex; **NR**: not reported; **OFC**: orbito-frontal cortex; **PCC**: posterior cingulate cortex; **PFC**: pre-frontal cortex; **R**: right; **SFG**: superior frontal gyrus; **vmPFC**: ventromedial prefrontal cortex

***** Main results are reported as differences between groups on resting-state functional connectivity

**References**

1. Shepherd AM, Matheson SL, Laurens KR, Carr VJ, Green MJ. Systematic Meta-Analysis of Insula Volume in Schizophrenia. Biological psychiatry. 2012;72(9):775-84.

2. Van Der Werff SJA, Pannekoek JN, Veer IM, Van Tol MJ, Aleman A, Veltman DJ, et al. Resting-state functional connectivity in adults with childhood emotional maltreatment. Psychological medicine. 2013;43(9):1825-36.

3. Thomason ME, Marusak HA, Tocco MA, Vila AM, McGarragle O, Rosenberg DR. Altered amygdala connectivity in urban youth exposed to trauma. Social cognitive and affective neuroscience. 2015;10(11):1460-8.

4. Fareri DS, Gabard-Durnam L, Goff B, Flannery J, Gee DG, Lumian DS, et al. Altered ventral striatal-medial prefrontal cortex resting-state connectivity mediates adolescent social problems after early institutional care. Development and psychopathology. 2017;29(5):1865-76.

5. Hoffmann F, Viding E, Puetz VB, Gerin MI, Sethi A, Rankin G, et al. Evidence for Depressogenic Spontaneous Thoughts and Altered Resting-State Connectivity in Adolescents With a Maltreatment History. Journal of the American Academy of Child and Adolescent Psychiatry. 2018;57(9):687-95.e4.

6. Pang Y, Zhao S, Li Z, Li N, Yu J, Zhang R, et al. Enduring effect of abuse: Childhood maltreatment links to altered theory of mind network among adults. Human brain mapping. 2022;43(7):2276-88.

7. Mattheiss SR, Levinson H, Rosenberg-Lee M, Graves WW. Exposure to violence is associated with decreased neural connectivity in emotion regulation and cognitive control, but not working memory, networks after accounting for socioeconomic status: a preliminary study. Behavioral and brain functions : BBF. 2022;18(1):15.

8. Sokołowski A, Kowalski J, Dragan M. Neural functional connectivity during rumination in individuals with adverse childhood experiences. European journal of psychotraumatology. 2022;13(1):2057700.

9. Gerin MI, Viding E, Puetz VB, Armbruster-Genc DJN, Rankin G, McCrory EJ. Atypical Interpersonal Problem-Solving and Resting-state Functional Connectivity in Adolescents with Maltreatment Experience. Current neuropharmacology. 2023.

10. Wang X, Liu Q, Fan J, Gao F, Xia J, Liu X, et al. Decreased functional coupling within default mode network in major depressive disorder with childhood trauma. Journal of psychiatric research. 2022;154:61-70.

11. Fan J, Gao F, Wang X, Liu Q, Xia J, Han Y, et al. Right amygdala-right precuneus connectivity is associated with childhood trauma in major depression patients and healthy controls. Social cognitive and affective neuroscience. 2023;18(1).

12. Schirmer ST, Beckmann FE, Gruber H, Schlaaff K, Scheermann D, Seidenbecher S, et al. Decreased functional connectivity in patients with major depressive disorder and a history of childhood traumatization through experiences of abuse. Behavioural brain research. 2023;437:114098.

13. Liu S, Fan D, He C, Liu X, Zhang H, Zhang H, et al. Resting-state cerebral blood flow and functional connectivity abnormalities in depressed patients with childhood maltreatment: Potential biomarkers of vulnerability? Psychiatry and clinical neurosciences. 2024;78(1):41-50.
